# Supplementary material for: Safety and Efficacy of Embolic Protection Devices in Saphenous Vein Graft Interventions: A Propensity Score Analysis—Multicenter SVG PCI PROTECTA Study
Source: J Clin Med. 2020 Apr 22;9(4):1198. doi: 10.3390/jcm9041198 (PMC7230434; doi:10.3390/jcm9041198)
Supplement: Supplementary file 1 [file jcm-09-01198-s001.pdf]

Journal of Clinical Medicine

MDPI

Table 1. 1-year follow up according to the use of embolic protection devices in the DES sub-group.

	EDPs (+)	EDPs (-)	Crude Calculation	
	n = 112	*n* = 267	HR (95% CI)	*p*-value
**MACCE**	19 (17.0)	62 (23.2)	0.68 (0.38-1.19)	0.177
**Death**	5 (4.5)	21 (7.9)	0.55 (0.20-1.49)	0.238
**MI**	5 (4.5)	19 (7.1)	0.61 (0.22-1.68)	0.338
**Stroke**	0 (0.0)	5 (1.9)	-	0.999
**TVR**	8 (7.1)	34 (12.7)	0.53 (0.24-1.18)	0.119
**TLR**	6 (5.4)	21 (7.9)	0.66 (0.26-1.69)	0.389

CI: confidence interval, EPDs- embolic protection devices, HR- hazard ratio, MACCE- major adverse cardiac and cerebrovascular events, MI- myocardial infarction, TLR- target lesion revascularization, TVR- target vessel revascularization.

Table 2. 1-year follow up according to the use of embolic protection devices in the no-thrombectomy sub-group.

	EDPs (+)	EDPs (-)	Crude Calculation	
	n = 180	*n* = 570	HR (95% CI)	*p*-value
**MACCE**	36 (20.0)	147 (25.8)	0.72 (0.48-1.09)	0.116
**Death**	7 (3.9)	43 (7.5)	0.50 (0.22-1.12)	0.092
**MI**	13 (7.2)	55 (9.6)	0.73 (0.39-1.37)	0.325
**Stroke**	2 (1.1)	10 (1.8)	0.63 (0.14-2.90)	0.552
**TVR**	16 (8.9)	70 (12.3)	0.70 (0.40-1.23)	0.697
**TLR**	12 (6.7)	50 (8.8)	0.74 (0.39-1.43)	0.743

CI: confidence interval, EPDs- embolic protection devices, HR- hazard ratio, MACCE- major adverse cardiac and cerebrovascular events, MI- myocardial infarction, TLR- target lesion revascularization, TVR- target vessel revascularization.

Table 3. Impact of the stent diameter on the 1-year follow up outcomes.

	HR	95%CI	*p*
**MACCE**	0.86	0.66-1.13	0.275
**Death**	0.83	0.52-1.33	0.443
**MI**	1.16	0.79-1.72	0.450
**Stroke**	1.06	0.41-2.70	0.908
**TVR**	0.80	0.55-1.16	0.237
**TLR**	0.83	0.54-1.27	0.390

Univariate logistic regression method was used. HR- hazard ratio, MACCE- major adverse cardiac and cerebrovascular events, MI- myocardial infarction, TLR- target lesion revascularization, TVR- target vessel revascularization.
